# The Lived Experience of Delirium in a Critically Ill Child: A Narrative Study

**DOI:** 10.1111/scs.70253

**Published:** 2026-04-17

**Authors:** Rikke Louise Stenkjaer, Ingrid Egerod, Mala Moszkowicz, Erwin Ista, Suzanne Forsyth Herling

**Affiliations:** ^1^ Department of Neonatal and Pediatric Intensive Care Copenhagen University Hospital Rigshospitalet København Denmark; ^2^ Department of Intensive Care Copenhagen University Hospital Rigshospitalet København Denmark; ^3^ Child and Adolescent Mental Health Center Copenhagen University Hospital København Denmark; ^4^ Department of Neonatal and Pediatric Intensive Care, Division of Pediatric Intensive Care, Erasmus MC‐Sophia Children's Hospital University Medical Center Rotterdam Rotterdam the Netherlands; ^5^ Department of Internal Medicine, Section of Nursing Science, Erasmus MC University Medical Center Rotterdam Rotterdam the Netherlands; ^6^ Department of Neuroanaesthesiology, Neuroscience Centre Copenhagen University Hospital Rigshospitalet København Denmark; ^7^ Department of Clinical Medicine University of Copenhagen København Denmark

**Keywords:** children, critical care nursing, family‐centred care, narrative approach, paediatric delirium, paediatric intensive care unit, qualitative research

## Abstract

**Background:**

While the negative experience of intensive care delirium has been established in adults, paediatric delirium (PD) is increasingly being recognized internationally. Knowledge, however, is still lacking regarding the experience of delirium in critically ill children. Therefore, we wish to provide insight into the subjective experience of delirium to better understand the children's perspective and enable the development of strategies to support children and their parents during and after the delirium episode.

**Aim:**

We aimed to describe the lived experience of delirium in a paediatric intensive care unit survivor. Our research question was: How does a child make sense of delirium after critical illness?

**Method:**

We interviewed 16‐year‐old Eric 10 months after discharge from the intensive care unit, using Frank's narrative theory to analyse Eric's narrative and performing thematic analysis to further interpret his experiences.

**Results:**

We identified Eric's story as a Quest narrative. According to Frank's theory, Eric uses his narrative to understand his experiences and perhaps to help others. We identified the following themes: Being lost, being pursued, being paralysed, and being back. During delirium, Eric lost control of reality during delusions and hallucinations. He experienced being pursued, being paralysed, and finally regaining his sense of reality. After discharge, Eric told and retold his story to his mother. Together, they developed a version of the story that was used to develop strategies to deal with delirium in the event of future admissions.

**Conclusion:**

Similarly to adults, children experience the distress of delusions and hallucinations during ICU delirium. The adolescent in our case was able to reflect on and suggest explanations for his delusional experiences. Mother and child prepared strategies to manage delirium during future admissions.

## 1 Background

Delirium is a neurocognitive disorder characterized by inattention and unawareness, with potential secondary cognitive changes [1]. The disorder often manifests with fluctuating severity as a direct consequence of a somatic illness [1]. Delirium is seen in all age groups and varies according to age, diagnosis, and condition of the patient [2, 3]. The estimated prevalence of paediatric delirium (PD) among critically ill children is 34% [3].

Critically ill children with delirium may experience discomfort and suffering, with symptoms such as disorientation, hallucinations, anxiety, altered behaviour and disturbed sleep‐wakefulness [1]. Delirium risk may present with predisposing and precipitating factors. Predisposing risk factors include young age, male sex, developmental delays and disabilities, pre‐existing medical conditions and overall illness severity [4, 5, 6]. Precipitating factors are associated with the treatment and environment in the paediatric intensive care unit (PICU), including mechanical ventilation, medications, infections, hypoxia, respiratory disorders, multiorgan failure, cardiac failure, physical restraints, noise and light [4, 5, 6]. Children with delirium are susceptible to increased length of hospital stay, more mechanical ventilator days, and overall increased healthcare expenses [4, 7, 8, 9]. Furthermore, an association between PD and lower quality of life after discharge has been shown [10]. It is reasonable to assume that PD may contribute to post‐intensive care syndrome (PICS) and repeated delusional memories in critical care child survivors [11, 12].

Experiences of delirium in adult intensive care unit (ICU) patients have been explored and described as distressing [13, 14]. Delirium recall in adults varies from total amnesia to vivid memories [15, 16]. Delusional memories often mix fact and fiction and are experienced as vivid, haunting, and lasting [17]. Fewer studies have investigated experiences of delirium in critically ill children. Some have used parents as the sources in the description of children's experiences [13, 18, 19, 20]. Delusional memories are reported in about a third of all children aged between 7 and 17 years admitted to the PICU, and the hypothesis that delusional memories would be positively associated with higher rates of post‐traumatic stress symptoms is confirmed [19]. Insight into the subjective delirium experience helps us to better understand the children's perspective, hence enabling the development of interventions to support children and their parents. This may reduce psychological morbidity and distress and support the development and implementation of delirium management for the improvement of clinical practice.

## 2 Aim

The aim of the present study was to describe the lived experience of delirium in a paediatric ICU survivor. Our research question was: How does a child make sense of delirium after critical illness?

## 3 Methodology

The study had a qualitative, descriptive and explorative design applying a narrative approach. Narratives, such as illness narratives, are described as stories that are told from the narrator's perspective, expressed in their own words and constructed to give meaning to their lived experiences [21]. Arthur Frank's narrative theory identifies three types of illness narratives: the restitution narrative (plot: I was healthy, I got ill, I became healthy again), the chaos narrative (non‐plot: life will never get better) and the quest narrative (plot: illness as motivation for change) [22, pp. 75–136]. The restitution narrative implies hope, whereas the chaos narrative demonstrates hopelessness. The quest narrative enables healing and a possibility of transformation as the narrator gains insight. Sharing a life story enables a clearer perspective on personal experiences. Telling the story is a way of purging or validating personal experience, which is central to the recovery process [23]. Arthur Frank introduced a story checklist during a workshop on narrative research to support analysis (Table 1). The checklist consists of eight core components: (1) Narrator, (2) Protagonist, (3) Complicating event or Trouble, (4) Helpers, (5) Antagonist, (6) Suspense, (7) Resolution and (8) Evaluation. The checklist helps to structure narratives to show how they convey meaning and emotion for the storyteller.

**TABLE 1:** Arthur Frank—Story checklist.

Narrator	Who speaks? Who tells the story?
Protagonist	How differs from narrator, e.g., time.
Complicating event or ‘trouble’	What happens that makes the story narratable, for whom?
Helpers	How do they make themselves known?
Antagonist	Who or what gets in the way, somehow opposing?
Suspense	Dramatic tension of opposition.
Resolution	What actions resolve Trouble, and how far is Trouble resolved?
Evaluation	What attitude is projected about what happened?

*Note:* Provided by Arthur Frank during a Narrative workshop in Copenhagen, December 2018.

### 3.1 Recruitment and Acquisition of Eric's Illness Narrative

The main participant in this study was an adolescent admitted to the Copenhagen University Hospital, Denmark. Eric (pseudonym) was admitted to the ICU, as the hospital did not have a designated paediatric ICU. While in ICU, Eric experienced delirium, which was confirmed by his mother, the bedside nurses and a child psychiatrist [24]. After Eric's latest hospitalization, his mother participated in a study exploring the parental perspective on PD [20]. Eric asked why he was not interviewed, as he was the one who had experienced delirium. We agreed that his story was important and interviewed Eric. This provided unique insight into the lived experience of delirium in a critically ill child, notwithstanding it was a single case narrative, and as such did not present a variation of experiences. At the time, we were unable to include more participants. This narrative, however, provides a good example of the issue at hand. Eric's illness narrative was recorded during a one‐hour interview at 10 months after his latest ICU discharge at his home as he preferred. His mother attended the interview to support her son. The interview was moderated by the primary investigator (PI) and a child psychiatrist who was part of the research team. We maintained a safe atmosphere that encouraged the child to tell his story and experiences of delirium in his own words in his own time. We did not use an interview guide but started with one opening question: ‘Would you like to tell us about your experiences with delirium when you were at the hospital?’ We posed follow‐up questions and used probes based on the narrative, non‐verbal communication, and short periods of silence. The interview was recorded digitally and transcribed verbatim. The two interviewers each took notes and debriefed after the interview. The notes served to elicit observations, supplement the dialogue, and enhance the reflection during the initial analysis [25].

### 3.2 Presentation of the Participants

The following description is composed by the authors based on an interview with Eric and an interview with his mother, including memos taken during the interviews and clinical knowledge and observations of the ICU environment.

Eric is a 16‐year‐old boy born with Crouzon syndrome and associated cranial deformation. He has been hospitalized more than 30 times for surgical correction of his deformities. Due to his illness, Eric has vision and hearing impairment and a psychiatric developmental disorder. He uses hand signals as a supplemental language. He lives with his parents and attends a school for children with special needs. Eric is particularly close to his mother, who reads aloud to him on his favourite subject, war history. He enjoys playing computer games with his friends, especially Fortnite, which is an online battle royale game developed by Epic Games in 2017 [26].

At present, Eric was admitted for scheduled facial surgery. After surgery, he was transferred to the ICU in a room with three adult patients. A workstation with a computer was at the end of the bed and an observation room for staff was located at the entrance to the room behind a glass partition. Nurses assessed Eric for delirium throughout his ICU stay. When he was delirious, his mother could always sense that something was very wrong. He was unable to recognize his mother and exhibited combative behaviour by lashing out at her and pulling at his tubes and lines. He was treated with antipsychotic medications and his mother managed to calm him by reading aloud from books, talking about their daily life, looking at family photos, and being present both day and night.

### 3.3 Analysis of the Illness Narrative

We used Frank's narrative theory and story checklist for initial analysis (Table 1), identifying Eric's story as a quest narrative, wherein the storyteller gains insight from the illness, rather than reaching recovery [22, 27]. This was evidenced by Eric's wish to tell his story so others could benefit from his experience of delirium. Eric's narrative did not have a clear chronologic structure, but we elicited four themes and connected them as a chronologic narrative. The themes were identified using thematic analysis as outlined by Braun and Clarke [21, 28]: reading the transcripts, generating codes, searching for themes, reviewing themes and naming the themes.

### 3.4 Ethical Considerations

The study was reported to the Danish Data Protection Agency (j.no.: P‐2019‐548) and reported to the National Committee on Health Research Ethics (reference number: 19076015). Eric and his mother were informed verbally and in writing about the aim of the study. Written informed consent was gained from both. They gave their permission to publish the narrative with quotes knowing that it appears not fully anonymized. We provided the contact details of the interviewer if the participants needed further debriefing after the interview [29].

## 4 Findings

We used the components in the Story Checklist to ensure the narrative structure of Eric's story: Eric was the narrator and protagonist. His mother was briefly a supporting narrator. The trouble Eric needed to overcome was delirium, where he lost contact with reality and lost track of his mother. Eric's mother was his helper who had told and retold the story with Eric as they tried to understand his delirious experiences. Loss of reality and confusion was the antagonist standing in the way of regaining a sense of reality while the suspense was whether Eric returned to reality and normal cognition. As a quest narrative, the story helped Eric to find meaning in his experiences and find solutions to resolve the trouble overcome delirium. Interestingly, Eric narrated his experiences by describing his delusions and adding running commentary and evaluation of what had actually happened. His ability to simultaneously describe and reflect on his experiences, suggested that he was familiar with telling his story. This also suggested that the story might have changed over time. Table 2 provides examples of the narrative analytical process.

**TABLE 2:** Examples of the narrative analytical process.

Core component	Description	Quote
Narrator	Eric is the storyteller	*Eric: ‘In reality, there was nothing. I felt that I heard things. I heard … In reality, I never saw these things, but I sensed what happened …’*
Protagonist	Eric is the main character	*Eric: ‘Yes, things went from Nordic mythology to cyber, each time I closed my eyes, it disappeared, and each time I opened them again, I was in a new situation. It was the olden days, and the French flag was in the ceiling … It was wartime …’*
Complicating event or ‘trouble’	Delirium is the trouble that must be resolved	*Eric: ‘About a monster in front of me? I couldn't see it, I got the feeling of it and was frightened, I got goosebumps … I didn't hear a sound, but I had the feeling that something looked at me, stared at me’*
Helpers	Eric's mother is the helper	*Eric: ‘Yes, I remember that I said, ‘Where is my mom’? And then you [Mother] said, ‘I'm here’ and you said that you were my mom, I remember that day … Until someone says Eric, and my last name, I always know that someone knows me, so know where I am…’*
Antagonist	The antagonist is loss of reality and loss of control	*Eric: ‘I could feel my legs, I could wiggle my toes, but I couldn't use them. One day, it was funny, my body thought I was hiding, but really, I sat and tried to get out of bed’*
Suspense	The suspense is whether Eric will return to reality and regain normal cognition	*‘…Until someone says Eric, and my last name, I always know that someone knows me, so know where I am. Then I can relax …’*

*Note:* The core components are provided by Arthur Frank during a Narrative workshop in Copenhagen, December 2018.

We identified the following four themes in Eric's narrative: Being lost; being pursued; being paralysed; and being back. Table 3 provides examples of the thematic analysis. The four themes constitute a chronological meta‐narrative of Eric's delirious experiences.

**TABLE 3:** Examples of the thematic analysis.

Quote	Codes	Theme
*Eric: ‘In reality, there was nothing. I felt that I heard things. I heard … In reality, I never saw these things, but I sensed what happened … In the beginning, it felt funny until I discovered that my mom wasn't there, and I didn't know anyone’*	Lost from reality Lost from his mother Loneliness Being afraid	Being lost
*Eric: ‘I didn't hear a sound or anything. I just had the feeling of someone looking at me, staring at me … I was prepared for the worst, I thought it was a doctor sitting there looking at me and looking at those things checking my heart, and … I felt I was kept under surveillance. They sat and watched me from the window or the ceiling* …’	Being watched by the nurses and physicians Surfaces which reflect images	Being pursued
*Eric: ‘The first night I felt that I was in a spaceship. The worst thing was when I tried to stand, I couldn't see who it was. I took the oximeter and used it as a light, I couldn't see anything and felt that I couldn't move my legs. I used the oximeter like a [torch] – that's why I didn't want to wear it’*	Unable to move Mixing up reality and dream	Being paralysed
*Eric: ‘Yes, then I'm watching YouTube, getting my brain on track again’*	How to manage delirium Back to reality	Being back

### 4.1 Being Lost

This theme described Eric's sensation of being lost and trying to find his bearings. He was lost from reality and his mother. He was bothered by hospital sounds and integrated these into his delirious experiences, where he believed that children in the unit broadcasted their problems in a loudspeaker. In his narrative, he confused technical devices, such as loudspeakers, microphones, call cords and wires with elements form everyday life.

Eric I heard a loudspeaker in my room. A loudspeaker that can be used to call outside. Some children who have a cord can call their mother. My god, two children used the loudspeaker and asked, ‘Where is Granddad’? ‘Where is he’? It was so irritating … They said, ‘god damn it, where's my mom’? They laughed and cried in the loudspeaker.

During delirium, Eric experienced auditory hallucinations but later, he reassured himself that the events were not real. Ten months passed since he was in ICU, and he has had time to reflect on what might have happened and may gradually have changed his narrative.

Eric In reality, there was nothing. I felt that I heard things. I heard … In reality, I never saw these things, but I sensed what happened … In the beginning, it felt funny until I discovered that my mom wasn't there, and I didn't know anyone.

### 4.2 Being Pursued

This theme described how Eric experienced being watched and pursued. Like dreams, delusions take their cues from reality [30]. Eric was monitored in the ICU; nurses were watching him closely, but his experience was more ominous, like undercover surveillance.

Eric I didn't hear a sound or anything. I just had the feeling of someone looking at me, staring at me … I was prepared for the worst, I thought it was a doctor sitting there looking at me and looking at those things checking my heart, and … I felt I was kept under surveillance. They sat and watched me from the window or the ceiling ….

The ICU environment was conducive to delirium. Many surfaces reflect images and lights made it eerie. At night Eric could see his reflection in the window and felt watched. The ceiling panels were shiny, enabling reflection. The workstation at the end of the bed projected blue light from computers and all the technical equipment was illuminated creating a strange environment. In this setting, Eric experienced paranoid delusions.

Eric About a monster in front of me? I couldn't see it, I got the feeling of it and was frightened, I got goosebumps … I didn't hear a sound, but I had the feeling that something looked at me, stared at me.

### 4.3 Being Paralysed

Eric described several experiences where he was unable to move because his legs felt paralysed. In reality, he was trapped in his bed, attached to various medical or technical devices. Eric's narrative included his delirious experiences and how he made sense of them later after he came home.

Eric The first night I felt that I was in a spaceship. The worst thing was when I tried to stand, I couldn't see who it was. I took the oximeter and used it as a light, I couldn't see anything and felt that I couldn't move my legs. I used the oximeter like a [torch] – that's why I didn't want to wear it.

Eric recalled an experience of being blinded and paralysed. Dream and reality were mixed when Eric used the pulse oximeter as a torch when he was delirious. When Eric was awake, he refused to wear the oximeter because he associated it with his delusional experiences.

Eric I was fooled into believing I was in a spaceship … When the light goes on, things get normal. The other side of the brain knows that I'm not on a spaceship, that I'm in Copenhagen … 95% of the brain thinks that I'm on a spaceship.

Eric's delusions were intensified when the room was dark. The experience of a spaceship was a paranoid delusion as his awareness fluctuated between the real world and delirious experiences. Eric described his fluctuating reality as two sides of the brain. On reflection, he tried to separate dreams from reality.

Eric I could feel my legs, I could wiggle my toes, but I couldn't use them. One day, it was funny, my body thought I was hiding, but really, I sat and tried to get out of bed.

Eric's delirious experiences included being a warrior in combat where the goal was survival. He had to hide to survive. In reality, his attempt to hide was an attempt to get out of bed and a battle for survival.

Eric Yes, things went from Nordic mythology to cyber, each time I closed my eyes, it disappeared, and each time I opened them again, I was in a new situation. It was the olden days, and the French flag was in the ceiling … It was wartime ….

Eric's delusional experiences were inspired by the Fortnite universe, where the characters struggle for survival in different settings and in different periods of time. Delirium distorted his thoughts, and he vividly experienced monsters and spaceships, thus explaining his combative behaviour.

### 4.4 Being Back

This theme described how Eric recalled being back in reality and trying to hold on to it. Eric experienced delirium (verified by psychiatrist) many times during his many surgeries. He and his mother developed ways to cope with delirium. One way to connect with reality was for Eric to recognize and feel the floor of the hospital room.

Eric I can recognize the hospital floor … so we have made a deal, that next time I'm delirious, they should turn on the lights so I can touch the floor with my feet, then I know where I am. I am at the [Rigshospitalet], my name is Eric, I was born in November 2006. Then I know, that's why we made the plan, every time I'm delirious, turn on the light, then I can feel the floor … the floor is like stone with sand.

The following description of Eric's reality might be generational at it shows contemporary interests of younger people. Eric says:

Eric Yes, then I'm watching YouTube, getting my brain on track again.

Eric described how he returned to reality. He needed to recognize his mother, and he needed someone to call his name as he was trying to maintain awareness of his own identity. This brought him back. He needed to have a structure and a plan for the near future.

Eric Yes, I remember that I said, ‘Where is my mom’? And then you [Mother] said, ‘I'm here’ and you said that you were my mom, I remember that day … Until someone says Eric, and my last name, I always know that someone knows me, so know where I am. Then I can relax … I remember that I lashed out at things. I always have a plan, if something happens, I always have someone call my name.

Eric's mother explained he had made a meticulous plan. His next surgery is planned for the fall. If he has trouble expressing something in words, he has developed some hand signals he can use.

Eric It's the place, every time I open the door, I can see, I always know where I am … I always take pictures – without knowing it, I take 30,000 pictures inside my head that I copy and check. Even if I'm in a new place, I go around and see where things are ….

Eric tried to record memories in his mind, memorizing signals that could help alleviate delusions in the future as he tried to find ways to recognize reality and escape his delusions.

## 5 Discussion

In the present study, we explored how a child might make sense of ICU delirium. We described the narrative structure of Eric's story by identifying the main components in the narrative. This helped us to describe the main themes constituting the chronological meta‐narrative.

In the theme ‘being lost’, Eric hoped to be found. Eric was an adolescent who spent much of his time with his mother. Children have been seen to regress to an earlier developmental step when they experience delirium [20]. From infancy, a child is able to separate from the caregiver when feeling safe. In insecure or dangerous situations, however, the child seeks closeness to the caregiver [31].

Eric had a feeling of being pursued and observed. These are common experiences in delirium in adults [32]. Eric tried to find plausible explanations for his experience of surveillance, e.g., nurses observed him in the ICU. He was spooked by reflections in the windows, which were later identified as his own reflection. The experiences were vivid, which is also often seen in delirious adults [17, 18]. Like dreams, delusions might include remnants of events of the day. Eric's delusions were influenced by events in the computer game Fortnite, which supports the description of children's hallucinations that often involve imaginative or playful elements, such as characters from their favourite stories or games [33, 34, 35]. Eric's use of familiar imaginative worlds can be understood through Frank's concept of the wounded storyteller, where the ill person creates meaning by drawing on culturally available narrative resources to domesticate overwhelming sensations [22]. Like adults, Eric thought he was fighting for his life [17]. Eric experienced being a wounded and paralysed warrior. In retrospect, this can be explained by sedative medications. Unreal experiences are often more vivid than dreams and may linger in affected adults for years [36].

In the theme ‘being paralysed’, Eric had visual hallucinations when he mistook his oximeter for a torch on a spaceship. Cognitive disturbances, including perceptual disturbances (delusions, hallucinations), are one of the core criteria in DSM‐V of delirium [1]. Hallucinations may be visual, tactile, or auditory [37]. Eric experienced various types of hallucinations. In the theme ‘being lost’, Eric had auditory hallucinations when he heard the other children broadcast their problems.

In the theme ‘being back’ Eric had suggestions as to how he could return to reality when he was delusional. He hoped that he would be able to manage delirium if it happened again. This might not be possible, but he believed that it would help if someone turned on the light, so he could see. He also believed that recognizing and feeling the hospital floor could reorient him to where he was. Finally, he wanted his mother nearby, so she could tell him where he was. Reorientation is part of the prevention and management of delirium in the ICU, but patients do not find this sufficient to help them out of the confusional state [14]. Physical contact and the presence of family are more important, which is similar to Eric's need for his mother's presence and assurance [14]. Family‐centred care is an approach emphasizing the individual child and family's preferences for care and treatment in partnership with healthcare professionals to reduce emotional distress and thereby potentially reduce the incidence and duration of delirium [38, 39].

Drawing on Frank's narrative theory, Eric's narrative can be understood not only as a clinical account of perceptual disturbance but as a struggle to restore coherence, identity and agency [22]. We were aware that the narrated experiences took place ten months earlier and that they would be influenced by telling and re‐telling his story, and reflection on the meaning of the experiences. Narratives are constructed by the narrator and reflect an attempt to revise or select details to give meaning to life experiences [21]. Stories are told to make meaning of events in life [21]. Eric discussed his experiences with his mother, and we assume that the narrative might have changed over time. It has been hypothesized that delusional memories might cause symptoms of posttraumatic stress [19]. We might also assume that this storytelling helped Eric to cope with his experiences, as he never developed posttraumatic stress.

## 6 Strengths and Limitations

The use of narrative theory and Eric's willingness to tell his story have increased the strength of our findings. The narrative theory provided a structured framework for understanding how and why Eric's story creates meaning. Eric increased our insight into how children might experience delirium with the mix of personal interests and the intensive care environment. We realize that a single narrative might limit our findings, but the narrative provides good insight into the situation, and the use of contextual data has strengthened the study. Although Eric is in some ways not age‐appropriate due to his psychiatric developmental disorder, we believe his story provides an informative view of the delirium experienced by an older child.

Credibility was increased by our use of a known method and by using investigator triangulation. Transferability was increased by describing the context, dependability by providing quotes, and confirmability by describing the sampling, data collection, and analysis [40]. The consolidated criteria for reporting qualitative research guidelines were used to ensure transparency of our reporting [41]. Data were organized and coded using the computer software NVivo Version 14 [42].

## 7 Conclusion

Our study generated knowledge to increase the understanding of the lived experience of delirium in a child. Like in adults, childhood delirium with delusions and hallucinations is distressing. Interestingly, the older child was able to reflect on the experiences and find plausible explanations for delusional encounters. Eric remembered repeating episodes of delirium and expects more to come as he will undergo surgeries in his life. Therefore, it was important for him to develop strategies in collaboration with close relatives for managing future episodes of delirium. Perhaps this could be explored further in future studies.

## Author Contributions

All authors are made substantial contributions to conception and design, or acquisition of data, or analysis and interpretation of data; Involved in drafting the manuscript or revising it critically for important intellectual content; Given final approval of the version to be published. Each author should have participated sufficiently in the work to take public responsibility for appropriate portions of the content; Agreed to be accountable for all aspects of the work in ensuring that questions related to the accuracy or integrity of any part of the work are appropriately investigated and resolved.

## Funding

This study was funded by the Copenhagen University Hospital, Rigshospitalet, Denmark, and the Novo Nordisk Foundation, Denmark (NNF 200OC0066074).

## Ethics Statement

This study was approved by the Danish Data Protection Agency (j.no.: P‐2019‐548) and reported to the National Committee on Health Research Ethics (reference number: 19076015).

## Consent

Participants were informed verbally and in writing about the aim of the study. Written informed consent was gained.

## Conflicts of Interest

The authors declare no conflicts of interest.

## Data Availability

The data that support the findings of this study are available on request from the corresponding author. The data are not publicly available due to privacy or ethical restrictions.
